# The extraordinary genus *Myja* is not a tergipedid, but related to the Facelinidae s. str. with the addition of two new species from Japan (Mollusca, Nudibranchia)

**DOI:** 10.3897/zookeys.818.30477

**Published:** 2019-01-23

**Authors:** Alexander Martynov, Rahul Mehrotra, Suchana Chavanich, Rie Nakano, Sho Kashio, Kennet Lundin, Bernard Picton, Tatiana Korshunova

**Affiliations:** 1 Zoological Museum, Moscow State University, Bolshaya Nikitskaya Str. 6, 125009 Moscow, Russia; 2 Reef Biology Research Group, Department of Marine Science, Faculty of Science, Chulalongkorn University, Bangkok 10330, Thailand; 3 New Heaven Reef Conservation Program, 48 Moo 3, Koh Tao, Suratthani 84360, Thailand; 4 Center for Marine Biotechnology, Department of Marine Science, Faculty of Science, Chulalongkorn University, Bangkok 10330, Thailand; 5 Kuroshio Biological Research Foundation, 560-I, Nishidomari, Otsuki, Hata-Gun, Kochi, 788-0333, Japan; 6 Natural History Museum, Kishiwada City, 6-5 Sakaimachi, Kishiwada, Osaka Prefecture 596-0072, Japan; 7 Gothenburg Natural History Museum, Box 7283, S-40235, Gothenburg, Sweden; 8 Gothenburg Global Biodiversity Centre, Box 461, S-40530, Gothenburg, Sweden; 9 National Museums Northern Ireland, Holywood, Northern Ireland, UK; 10 Queen’s University, Belfast, Northern Ireland, UK; 11 Koltzov Institute of Developmental Biology RAS, 26 Vavilova Str., 119334 Moscow, Russia

**Keywords:** Facelinidae, morphological data, molecular phylogeny, *
Myja
*, new species, Nudibranchia, taxonomy, West Pacific Ocean

## Abstract

Morphological and molecular data are presented for the first time in an integrative way for the genus *Myja* Bergh, 1896. In accordance with the new molecular phylogenies, the traditional Facelinidae is paraphyletic. Herein is presented the phylogenetic placement of true Facelinidae s. str., including the first molecular data for *F.auriculata* (Müller, 1776), type species of the genus *Facelina* Alder & Hancock, 1855. The taxonomic history of *F.auriculata* is reviewed. The genus *Myja* is related to the clade Facelinidae s. str., but shows disparate morphological traits. Two new species of the genus *Myja*, *M.karin***sp. n.**, and *M.hyotan***sp. n.**, are described from the Pacific waters of Japan (middle Honshu), and M.cf.longicornis Bergh, 1896 is investigated from Thailand. According to molecular analysis and review of available morphological information, the genus *Myja* contains more hidden diversity. The family-level relationship within aeolidacean nudibranchs with emphasis on the family Facelinidae is outlined. The problem of the relationship between Facelinidae Bergh, 1889 and Glaucidae Gray, 1827 is discussed. The family Glaucidae has precedence over Facelinidae and is phylogenetically related to the core group of Facelinidae s. str., but has a profoundly modified aberrant external morphology, thus making a purely molecular-based approach to the taxonomy an unsatisfactory solution. To accommodate recently discovered hidden diversity within glaucids, the genus *Glaucilla* Bergh, 1861 is restored. The family Facelinidae s. str. is separate from, and not closely related to, a clade containing the genera *Dondice* Marcus, 1958, *Godiva* MacNae 1954, *Hermissenda* Bergh, 1879, and *Phyllodesmium* Ehrenberg, 1831 (= *Myrrhine* Bergh, 1905). The oldest valid available name for the separate ex-facelinid paraphyletic clade that contains several facelinid genera is Myrrhinidae Bergh, 1905, and resurrection of this family name under provision of the ICZN article 40.1 can preliminarily solve the problem of paraphyly of the traditional Facelinidae. “Facelinidae” s. l. needs to be further divided into several separate families, pending further study.

## Introduction

The genus *Myja* Bergh, 1896 was described more than one century ago (Bergh 1896) and since then has never been re-described, nor phylogenetically assessed. It was originally referred to the family Tergipedidae by Bergh (1896), most likely due to some external similarities to the genus *Tergipes*. The morphological characters of the genus *Myja*, an acleioproctic anus in combination with club-shaped cerata that mimic its prey and a diminutive uniserial radula, make taxonomic assessment of this extraordinary-looking genus difficult. In the present study, we obtained recently collected specimens from the Indo-West Pacific tropics (Thailand) for the first time. These are very similar by general external and internal patterns to the type species of the genus *Myja*, *M.longicornis* Bergh, 1896 that was described from the Indo-West Pacific island of Ambon, but also show some fine-scale differences which prevent us from concluding that the Thai specimens belong to the type species of the genus. However, the unique morphological similarity between type species of the genus *M.longicornis* and our material unambiguously allows it to be included in the genus *Myja* and thus reveals the molecular phylogeny of one of the most enigmatic nudibranchs. Additionally, specimens were obtained that are externally and internally similar to the genus *Myja*, from the Pacific coast of the main Japanese island Honshu. The *Myja* from Thailand is shown to be morphologically and genetically distinct from the Japanese and all three species are described here. Furthermore, our molecular phylogenetic analysis shows that the genus *Myja* is unrelated to the family Tergipedidae, contrary to the opinion of Bergh (1896), but instead it is part of the traditional Facelinidae family. Because the family Facelinidae is composed of a large morphological and molecular assemblage (e.g., Miller 1974; Millen and Hermosillo 2012; Korshunova et al. 2017a) the phylogenetic position of the family is also tested after inclusion of the novel molecular data on *Myja*. Previously it has been shown that the traditional Facelinidae is paraphyletic (e.g., Goodheart et al. 2017, 2018) but in the absence of molecular data on the type species of the genus *Facelina* Alder & Hancock, 1855, the position of Facelinidae s. str. was uncertain. In this study we present the first molecular data for *F.auriculata* (Müller, 1776), the type species of the genus *Facelina*, and therefore we are able to identify the group of taxa that relates to Facelinidae s. str. The present analysis is corroborated by previous results (Goodheart et al. 2017, 2018), and confirms that the family Facelinidae is paraphyletic and needs to be separated into several smaller families.

## Materials and methods

### Collecting data

Three specimens of two new Japanese species were collected by SCUBA diving in the Pacific coast of Japan (Honshu, Osezaki) by Tatiana Korshunova, Alexander Martynov, and Hiroshi Takashige. Three specimens of Myjacf.longicornis were collected by SCUBA diving in Thailand waters by Rahul Mehrotra and Suchana Chavanich. Additional facelinid specimens were collected in UK, Norway, Sweden, and at the Sea of Japan. All specimens were preserved in 80–95% EtOH.

### Morphological analysis

**Figure 1.**
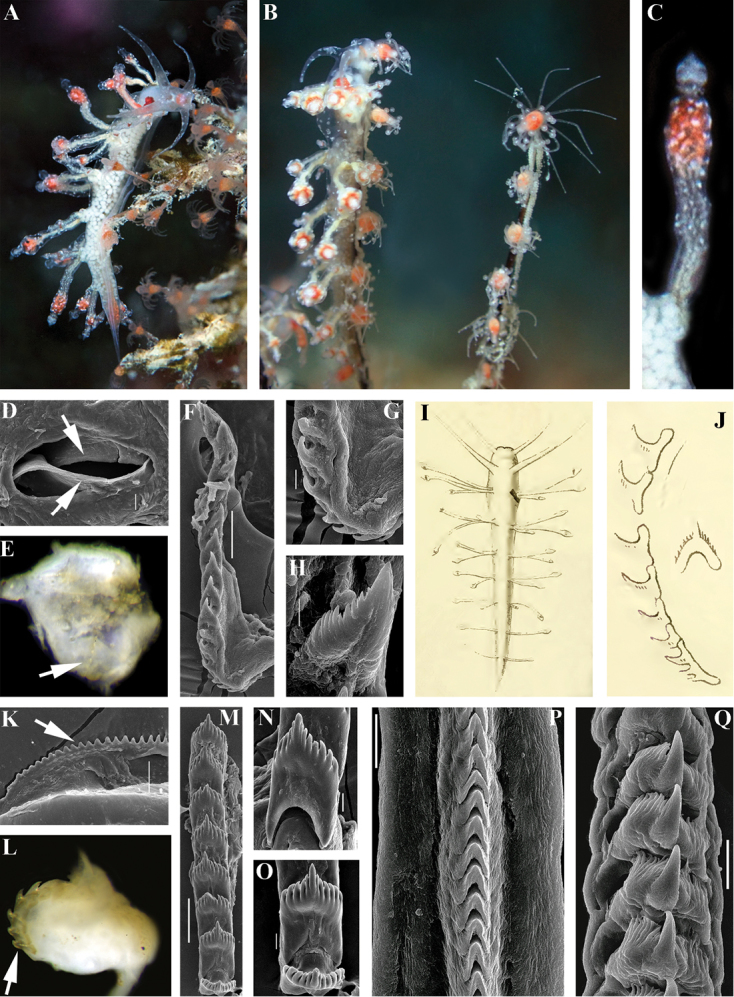
Comparison of *Myjalongicornis* Bergh, 1896 with other aeolidacean taxa that have been proposed to have relationships with it (*Calma*, *Tergipes*) and which are covered by present analysis [type species of the genus *Facelina*, *F.auriculata* (Müller, 1776)]. **A–H**Myjacf.longicornis from Thailand, living animal ca. 10 mm in length **A** dorsal view of hydroids in situ **B** lateral view of hydroids in situ (left), egg mass on the hydroid (right) **C** details of cerata **D** smooth masticatory processes of jaws (indicated by arrows), SEM **E** pharynx, dissected dorsally to show very narrow radula (indicated by an arrow), LM **F** whole radula, SEM **G** anteriormost part of radula to show sacoglossan-like small knife-shaped teeth, SEM **H** teeth from the middle part of radula, SEM; **I, J***Myjalongicornis* Bergh, 1896 external view and radula (anterior part), reproduced from the first description by Bergh (1896); **K–O***Facelinaauriculata* jaws and radula of a specimen from UK, collected together with neotype **K** masticatory process (well-defined denticles indicated by arrow), SEM **L** radula (arrow) on odontophore, to show that anteriormost teeth are not reduced, LM **M** anterior part of radula to show that teeth are not reduced **N** anteriormost tooth of radula **O** two anterior teeth of radula **P** radula (middle part) of *Calmaglaucoides* (Alder & Hancock, 1854) from Norway **Q** radula (middle part) *Tergipestergipes* (Forsskål in Niebuhr, 1775). Scale bars: 20 μm (**D, N, O, Q**); 50 μm (**F, K**); 10 μm (**G, P**); 5 μm (**H**); 100 μm (**M**). Photographs of living specimens by Chanon Ngernthongdee and Siwat Worachananant, SEM images by AV Martynov. Figures I and J are reproduced from Bergh (1896), the publication not currently under copyright.

**Figure 2.**
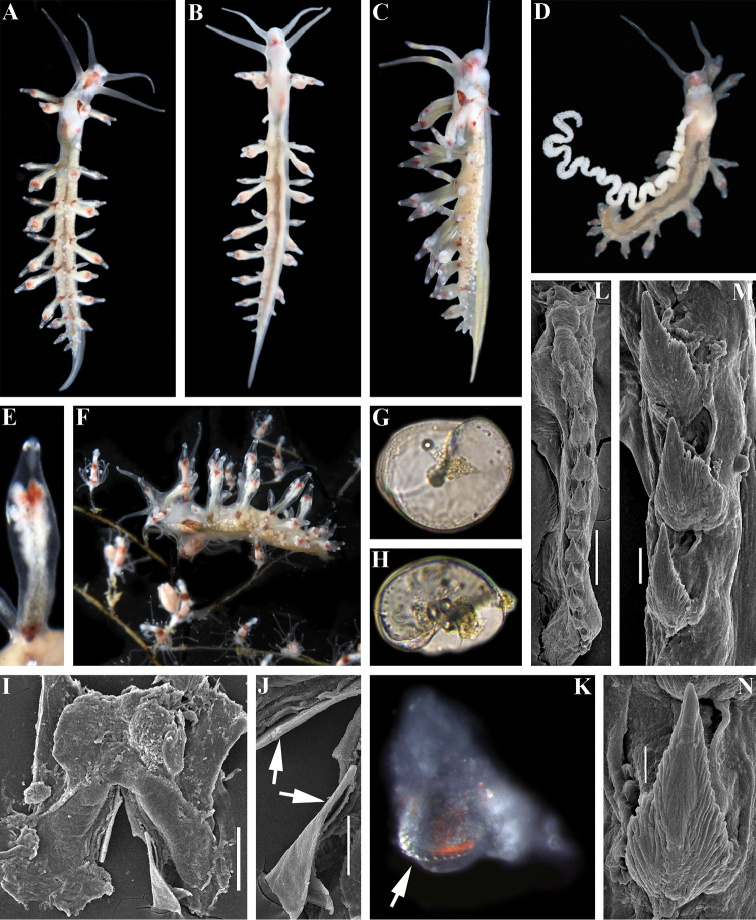
*Myjakarin* sp. n. **A–D** holotype **A** dorsal view **B** ventral view **C** lateral view **D** animal with egg mass **E** details of cerata **F** lateral view on hydroids in situ **G, H** veligers; **I–N** paratype **I** jaws **J** smooth masticatory processes of jaws (inidicated by arrows), SEM **K** radula on odontophore, to show narrow teeth and reduced anteriormost teeth (arrow), LM **L** whole radula, SEM **M** teeth from the middle part of radula **N** anterior teeth. Scale bars: 100 μm (**I**); 50 μm (**J, L**); 10 μm (**M**); 5 μm (**N**). Photographs of living specimens by TA Korshunova and AV Martynov, SEM images by AV Martynov.

**Figure 3.**
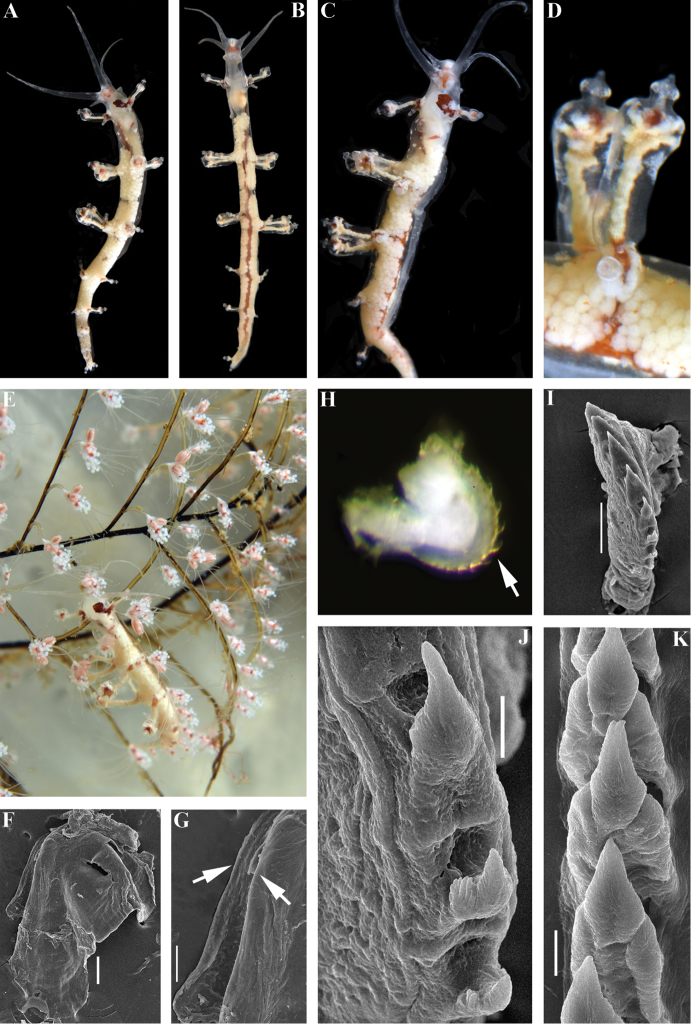
*Myjahyotan* sp. n., holotype. **A** dorsal view **B** ventral view **C** lateral view **D** details of cerata **E** dorsal view on hydroids in situ **F** jaw **G** smooth masticatory processes of jaws (indicated by arrows), SEM **H** radula on odontophore, to show reduced anteriormost teeth (arrow), LM **I** anterior teeth with strongly reduced anteriormost teeth, SEM **J** teeth from the middle part of radula **K** posterior part of radula to show smooth teeth. Scale bars: 100 μm (**F**); 50 μm (**G, I**); 10 μm (**J, K**). Photographs of living specimens by TA Korshunova and AV Martynov, SEM images by AV Martynov.

**Figure 4.**
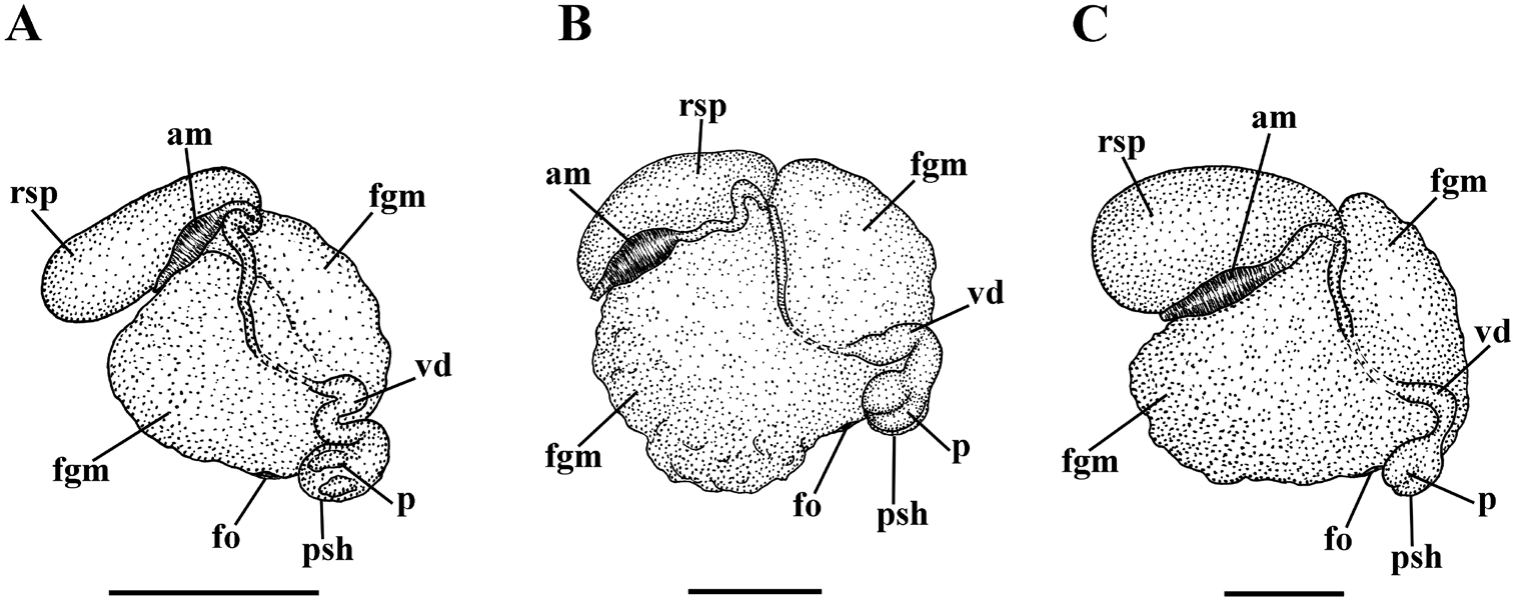
Reproductive systems of new species of the genus *Myja*. **A**Myjacf.longicornis**B***Myjakarin* sp. n. **C***Myjahyotan* sp. n. Abbreviations: am – ampulla; fgm – female gland mass; fo – female opening; p – penis; psh – penial sheath; rsp – proximal receptaculum seminis; vd – vas deferens.

All specimens were examined with a stereomicroscope (MBS-9) and photographed using Nikon D-90 and D-810 digital cameras with a set of extension rings. The pharynxes were removed and processed with a weak solution of domestic bleach (NaClO). The jaws were examined using a stereomicroscope and digital cameras. The jaws and radulae were examined under a scanning electron microscope (JSM and CamScan Series II) (Figs 1–4).

### Molecular analysis (Fig. 5)

Specimens of *Myja* from Japan and Thailand were sequenced for the mitochondrial genes cytochrome c oxidase subunit I (COI) and 16S rRNA, and the nuclear gene Histone 3 (H3). Additionally, one specimen of *Facelinaauriculata* from the UK was sequenced. DNA extraction procedure, PCR amplification options, and sequence obtainment have been previously described in detail (Korshunova et al. 2017a, b; 2018a). Protein-coding sequences were translated into amino acids for confirmation of the alignment. All new sequences were deposited in GenBank (Table 1, highlighted in bold). Publicly available sequences of representatives of the suborder Aeolidacea, plus several outgroup taxa (*Tritonia*, *Dendronotus*, *Bonisa*, and *Janolus*) were also included in the molecular analysis. Sequences were aligned with the MAFFT algorithm (Katoh et al. 2002). Separate analyses were conducted for COI (657 bp), 16S (471 bp), H3 (327 bp), and concatenated data (1455 bp). Evolutionary models were selected using MrModelTest 2.3 (Nylander et al. 2004). Two different phylogenetic methods, Bayesian inference (BI) and Maximum Likelihood (ML), were used to infer evolutionary relationships. Bayesian estimation of posterior probability was performed in MrBayes 3.2 (Ronquist et al. 2012). Four Markov chains were sampled at intervals of 500 generations. Analysis was started with random starting trees and 10^7^ generations. Maximum likelihood-based phylogeny inference was performed in RAxML 7.2.8 (Stamatakis et al. 2008) with bootstrap in 1000 pseudo-replications. Final phylogenetic tree images were rendered in FigTree 1.4.2 (http://tree.bio.ed.ac.uk). Alignment from the 16S of *Myja* specimens was processed in Automatic Barcode Gap Discovery (ABGD, available at http://wwwabi.snv.jussieu.fr/public/abgd/abgdweb.html) with the following settings: a prior for the maximum value of intraspecific divergence between 0.001 and 0.1, 10 recursive steps within the primary partitions defined by the first estimated gap, and a gap width of 0.8. 16S alignment was analysed separately using both proposed models Jukes-Cantor (JC69) and Kimura (K80). The program Mega7 (Kumar et al. 2016) was used to calculate the uncorrected p-distances.

**Table 1.** List of samples, localities, and voucher references. The species in bold font are those sequenced in this study.

Species	Voucher, Locality	COI	16S	H3
*Aeolidiacampbellii* (Cunningham, 1871)	ZSM 20020700 Chile	KF317849	KF317837	KF317859
*Aeolidiafilomenae* Kienberger, Carmona, Pola, Padula, Gosliner & Cervera, 2016	MNCN:15.05/74477 France	KU160588	KU160562	KU160606
*Aeolidialoui* Kienberger, Carmona, Pola, Padula, Gosliner & Cervera, 2016	MNCN:15.05/74483 Oregon, USA	KU160591	KU160565	KU160607
*Aeolidiapapillosa* (Linnaeus, 1761)	ZMMU:Op-559 Russia	KX758257	KX758252	KX758261
*Aeolidiellaglauca* (Alder & Hancock, 1845)	ZMMU Op-560 Norway	KX758255	KX758254	KX758259
*Anteaeolidiellacacaotica* (Stimpson, 1855)	CASIZ174212 Line Islands	JQ997030	JQ996825	JQ996926
*Aeolidiellasanguinea* (Norman, 1877)	MNCN/ADN51933 France	JX087537	JX087465	JX087599
*Amphorinaodhneri* (Derjugin & Gurjanova, 1926)	ZMMU:Op-484 Russia	MF523318	MF523396	MF523244
*Amphorinapallida* (Alder & Hancock, 1842)	GNM9094 Scotland	KY129030	KY128821	KY128616
*Bohuslaniamatsmichaeli* Korshunova, Lundin, Malmberg, Picton & Martynov, 2018	ZMMU:Op-600 Sweden	MG323542	MG323548	MG323563
*Borealeanobilis* (A. E. Verrill, 1880)	ZMMU:Op-510 Russia	MF523347	MF523411	MF523271
*Bulbaeolidiajaponica* (Eliot, 1913)	CASIZ184527 Japan	JQ997033	JQ996828	JQ996929
*Bonisanakaza* Gosliner, 1981	CASIZ176146 South Africa	HM162746	HM162670	HM162579
*Calmaglaucoides* (Alder & Hancock, 1854)	ZMMU:Op-603 Norway	MG323544	MG323550	MG323565
*Catrionaaurantia* (Alder & Hancock, 1842)	ZMMU:Op-545 Norway	KY985467	MF523458	MG386404
*Cerberillabernadettae* Tardy (1965)	MNCN/ADN51957 Spain	JX087555	JX087489	JX087625
*Coryphellaverrucosa* (Sars M., 1829)	ZMMU:Op-521 Russia	MF523375	MF523421	MF523300
*Cratenaminor* Padula, Araújo, Matthews-Cascon & Schrödl, 2014	ZSM:Mol:20110345 Brazil	KJ940476	–	KM079346
*Cratenaminor* Padula, Araújo, Matthews-Cascon & Schrödl, 2014	ZSM Mol 20110338a Brazil	KJ940477	–	KM079341
*Cratenaminor* Padula, Araújo, Matthews-Cascon & Schrödl, 2014	ZSM Mol 20110338b Brazil	KJ940478	–	KM079342
*Cratenaperegrina* (Gmelin, 1791)	ZSM Mol 20020957 France	KJ940481	–	KM079349
*Cratenaperegrina* (Gmelin, 1791)	ZSM Mol 20100125 Croatia	KJ940480	–	KM079347
*Cratenaperegrina* (Gmelin, 1791)	MNCN15.05/53691 Senegal	HQ616752	HQ616715	–
*Cuthonanana* (Alder & Hancock, 1842)	ZMMU:Op-522 Russia	MF523376	MF523397	MF523301
*Cuthonellasoboli* Martynov, 1992	ZMMU:Op-524 Russia	MF523378	MF523457	MF523303
*Diaphoreolisviridis* (Forbes, 1840)	ZMMU:Op-537 Russia	MG266028	MG266026	MG266029
*Dendronotusdalli* Bergh, 1879	ZMMU:Op-295 Russia	KM397001	KM397083	KM397094
*Dendronotuslacteus* (W Thompson, 1840)	ZMMU:Op-286 Russia	KC660034	KC611290	KC660050
*Dendronotusrobustus* AE Verrill, 1870	ZMMU:Op-391 Russia	KM396970	KM397053	KM397120
*Dondiceoccidentalis* (Engel, 1925)	LACM2003-41.5	JQ699570	JQ699482	JQ699394
*Eubranchustricolor* Forbes, 1838	ZMMU:Op-525 Norway	MF523379	MF523399	MF523304
***Facelinaauriculata* (Müller, 1776)**	**ZMMU:Op-669 UK**	**MK320904**	**MK320915**	–
*Facelinabostoniensis* (Couthouy, 1838)	CAS184184 New Hampshire	KY129046	KY128837	KY128632
*Facelinavicina* (Bergh, 1882)	GNM Gastropoda 9310 Croatia	KY513634	KY513630	–
Facelinidae sp. 2	CASIZ186258 Philippines	JQ997075	JQ996879	JQ996984
*Favorinusbranchialis* (Rathke, 1806)	MNCN15.05/53695 Spain	HQ616761	HQ616724	HQ616790
*Favorinuselenalexiae* Garcia & Troncoso, 2001	CASIZ178875 Costa Rica	HM162755	HM162679	HM162588
*Favorinustsuruganus* Baba & Abe, 1964	CASIZ 186044 Philippines	JX220450	JX220482	JX220418
*Fionapinnata* (Eschscholtz, 1831)	CASIZ 088586 USA	KU757491	KU757615	KU757600
*Fjordialineata* (Lovén, 1846)	ZMMU:Op-508 Norway	MF523345	MF523404	MF523269
*Janoluslongidentatus* Gosliner, 1981	CASIZ176320 South Africa	HM162749	HM162673	HM162582
*Glaucusatlanticus* Forster, 1777	NM:W7469 Indian	JQ699603	JQ699517	JQ699429
*Glaucusatlanticus* Forster, 1777	UMMZ302975 North Atlantic	JQ699574	JQ699488	JQ699400
*Glaucillamarginata* Reinhardt & Bergh, 1864	CASIZ176985 Indian	JQ699604	JQ699518	JQ699430
*Glaucillamarginata* Reinhardt & Bergh, 1864	CASIZ176985 Indian	JQ699605	JQ699519	JQ699431
*Godivaquadricolor* (Barnard, 1927)	CASIZ176385 South Africa	HM162756	HM162680	HM162589
*Guleniamonicae* Korshunova, Martynov, Bakken, Evertsen, Fletcher, Mudianta, Saito, Lundin, Schrödl & Picton, 2017	ZMMU:Op-408 Norway	MF523373	MF523441	MF523297
*Hermissendacrassicornis* (Eschscholtz, 1831)	CPIC01115 Canada	KU950178	KU950121	KU950212
*Hermissendaopalescens* (J. G. Cooper, 1863)	CPIC00565 USA, California	KU950191	KU950126	KU950220
*Himatinatrophina* (Bergh, 1890)	ZMMU:Op-532 Russia	MF523389	MF523460	MF523314
*Itaxiafalklandica* (Eliot, 1907)	ZSM Mol-20070592 Chile	MF523334	MF523467	MF523258
*Luisellababai* (Schmekel, 1972)	MNCN15.05/53698 Spain	HQ616783	HQ616754	HQ616717
*Microchlamyllagracilis* (Alder & Hancock, 1844)	ZMMU:Op-503 Norway	MF523338	MF523444	MF523262
*Murmaniaantiqua* Martynov, 2006	ZMMU:Op-399 Russia	MF523390	MF523394	MF523315
***Myjakarin* sp. n.**	**ZMMU:Op-610 Japan**	**MK320900**	**MK320910**	**MK320905**
***Myjakarin* sp. n.**	**ZMMU:Op-611 Japan**	**MK320901**	**MK320911**	**MK320906**
***Myjahyotan* sp. n.**	**ZMMU:Op-612 Japan**	–	**MK320912**	**MK320907**
**Myjacf.longicornis Bergh, 1896**	**ZMMU:Op-667 Thailand**	**MK320902**	**MK320913**	**MK320908**
**Myjacf.longicornis Bergh, 1896**	**ZMMU:Op-668 Thailand**	**MK320903**	**MK320914**	**MK320909**
*Occidenthellaathadona* (Bergh, 1875)	ZMMU:Op-498 Russia	MF523332	MF523414	MF523256
*Orienthellatrilineata* (O’Donoghue, 1921)	CAS179466 California	KY129064	KY128855	KY128649
*Phyllodesmiumtuberculatum* Moore & Gosliner, 2009	CASIZ 177520 Philippines	HQ010490	HQ010525	HQ010457
*Phyllodesmiumjakobsenae* Burghardt & Wägele, 2004	CASIZ 177576 Philippines	HQ010489	HQ010524	HQ010456
*Sakuraeolisjaponica* (Baba, 1937)	MABIK MO0015762 Korea	KX610997	KX610997	–
*Sakuraeolisenosimensis* (Baba, 1930)	CASIZ178876 USA, California	HM162758	HM162682	HM162591
*Samlatakashigei* Korshunova, Martynov, Bakken, Evertsen, Fletcher, Mudianta, Saito, Lundin, Schrödl & Picton, 2017	ZMMU:Op-530 Japan	MF523384	MF523463	MF523309
*Tenelliaadspersa* (Nordmann, 1845)	CAS184191 New Hampshire	KY129085	KY128876	KY128668
*Tergipestergipes* (Forsskål in Niebuhr, 1775)	WS3463 Barents Sea	KY129090	KY128881	–
*Trinchesiacaerulea* (Montagu, 1804)	ZMMU:Op-622 Norway	MG266024	MG266022	MG266025
*Tritonianilsodhneri* Marcus Ev., 1983	CASIZ176219 South Africa	HM162716	HM162641	HM162548
*Tritoniaplebeia* Johnston, 1828	ZMMU:Op-572 Norway	KX788134	KX788122	–
*Zelentianinel* Korshunova, Martynov & Picton, 2017	ZMMU:Op-509 Russia	KY952178	MF523400	MF523242
*Zeusiahyperborea* Korshunova, Zimina & Martynov, 2017	ZMMU:Op-557 Russia	KX758256	KX758251	KX758260

## Results

### Taxonomy and molecular analysis

**Figure 5.**
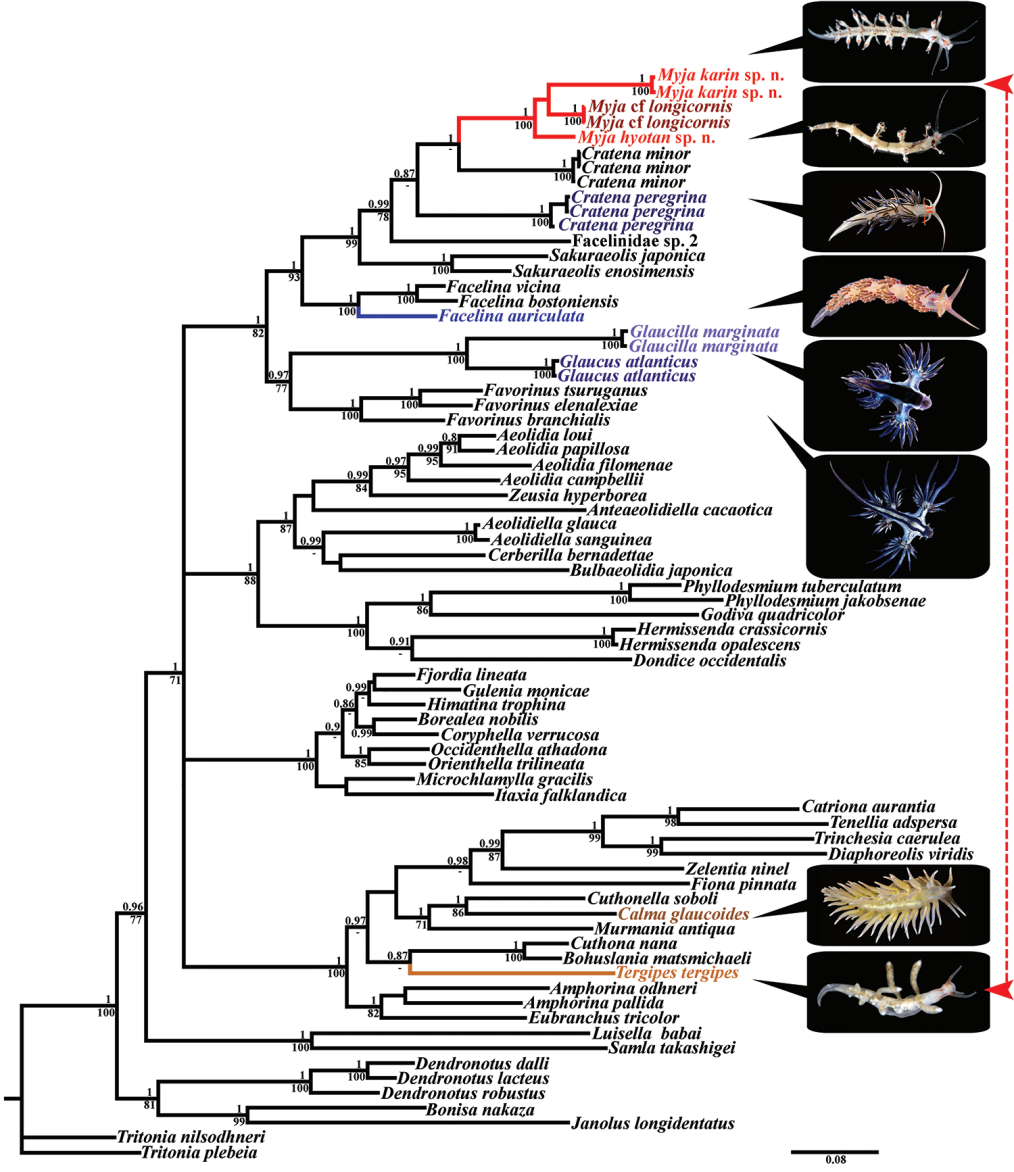
Phylogenetic tree of aeolidacean nudibranchs based on concatenated molecular data (COI + 16S + H3) represented by Bayesian Inference (BI). Numbers above branches represent posterior probabilities from Bayesian Inference. Numbers below branches indicate bootstrap values for Maximum Likelihood. The key clades and illustrated taxa are highlighted in colour. Two taxa with highly convergent external morphology but very distantly related according to the molecular analysis, the Tergipedidae and the genus *Myja*, are connected by a dotted red line. Neotype ZMMU Op-669 of *Facelinaauriculata* (Müller, 1776) is illustrated on the tree (photograph BE Picton).

The molecular analysis revealed and confirmed the position of the genus *Myja* as not related to the family Tergipedidae, but instead belonging to the Facelinidae s. str. “superclade” (Fig. 5). The part of the traditional “Facelinidae” including genera *Dondice*, *Godiva*, *Hermissenda*, and *Phyllodesmium* in turn show strong paraphyly and are distantly related to the Facelinidae s. str. (Fig. 5). Phylogenetic analysis was performed using five specimens of the genus *Myja*, sixty-one representatives of the suborder Aeolidacea, and seven outgroup specimens. The GTR model was chosen for the combined dataset for the mitochondrial COI and 16S and the nuclear H3. Bayesian Inference (BI) and Maximum Likelihood (ML) analyses based on the combined dataset yielded similar results (Fig. 5).

Molecular phylogenetic analyses among other important results also revealed phylogenetic positions of the type taxon *Facelinaauriculata*, and the taxa *Glaucus* and *Glaucilla* within the proper Facelinidae s. str. “superclade” (Fig. 5) (see the Discussion for details).

### Family Facelinidae s. str.

#### Myja

Taxon classificationAnimaliaNudibranchiaTergipedidae

Bergh, 1896

##### Type species.

*Myjalongicornis* Bergh, 1896.

##### Diagnosis.

One pair of anterior rows of cerata, posterior cerata in rows, few (1–3) peculiar club-shaped cerata per row, anus acleioproctic, rhinophores smooth, oral tentacles present, no anterior foot corners, cnidosacs present, pharynx moderately broad, jaws with wing-shaped anterior expansion, smooth masticatory edges, radula very small, uniserial, radular teeth very narrow, triangular with strong cusp, lateral denticles small, penis unarmed, supplementary glands absent.

##### Species included.

Myjacf.longicornis (Thailand), *Myjakarin* sp. n. (Japan), *Myjahyotan* sp. n. (Japan).

##### Remarks.

All *Myja* specimens studied here clustered together (PP = 1, BS = 100) in a maximum-supported clade. This agrees well with the results of the morphological analysis. Inside the *Myja* clade clustered maximum-supported (PP = 1, BS = 100) Myjacf.longicornis and *M.karin* sp. n. clades and *M.hyotan* sp. n. clade. The ABGD analysis of the 16S data set run with two different models revealed three potential species: Myjacf.longicornis, *M.karin* sp. n., and *M.hyotan* sp. n. Additionally, molecular phylogenetic analysis revealed that *Cratenaperegrina* (Gmelin, 1791) and *Cratenaminor* Padula, Araújo, Matthews-Cascon & Schrödl, 2014 specimens clustered together on two maximum-supported (PP = 1, BS = 100) clades, which are not sister to each other. Furthermore, the *Cratenaminor* clade is sister to the *Myja* clade but without high node support (PP = 1, BS = 68). It is assumed that further analysis with the addition of a larger number of species and genes will clarify the phylogenetic relationship in *Cratena* species and may reveal hidden paraphyly of the genus *Cratena*. It is important to note that in Padula et al. (2014), it is shown that the *Sakuraeolisenosimensis* clade was wedged between the *C.minor* and *C.peregrina* clades in the Maximum Likelihood phylogenetic tree based on H3 sequences. The morphological and molecular differences for the known *Myja* species are included below.

#### Myja cf. longicornis

Taxon classificationAnimaliaNudibranchiaTergipedidae

Bergh, 1896

Figs 1 , 4

##### Material.

1 specimen, ZMMU Op-667, 6 mm long (fixed), Thailand, Koh Samaesan, 21 June 2018, depth 8 –16 m, soft sediment habitats, hydroids, collectors Rahul Mehrotra, Suchana Chavanich. 2 specimens, ZMMU Op-668, ca. 3 and 2 mm (fixed) same locality and collectors.

##### Locality.

Thailand, Chonburi, Koh Samaesan.

##### Diagnosis.

Up to eight ceratal rows, ground colour translucent greyish, ceratal cores light to dark greyish, ceratal tops dull reddish, apices with white spot, anterior cerata with prominent reddish basal spot (distributed over the whole surface in some cerata), white gonad spherules moderately dense, sparse white spots in the first half of the dorsal part, cerata moderately widened at top without smaller separate cupola-shaped tip, central tooth with sharp to pitted top and numerous lateral denticles, up to 23 small denticles, irregular in size, no distinct furrows and ridges on the teeth surfaces, no accessory penial gland, penis unarmed.

##### Description.

Body very elongate, up to 6 mm in preserved length (up to 10 mm alive) (Fig. 1A, B). Rhinophores similar in size to oral tentacles, smooth. Dorsal papillae cylindrical, forming eight ceratal rows along dorsal edges. Apices of papillae moderately to slightly widened, without cupola-shaped appendage (Fig. 1C). Notal edge absent. Anal opening acleioproctic on right side before first posterior ceratal rows. Reproductive openings lateral, below first anterior and second posterior rows of cerata. Ground colour translucent greyish. Oral tentacles and rhinophores with scattered opaque white dots. Digestive gland in the cerata (ceratal cores) light to dark greyish, digestive gland in upper part of cerata with reddish internal spot, apices with white spot. Anterior cerata with prominent reddish basal spot in some cerata distributed over its whole surface. Central branches of digestive gland visible through dorsal part of body greyish. Numerous small, moderately dense white gonads appear as white spherules that shine through dorsal surface. Jaws broadly triangular with prominent anterior wings, masticatory borders smooth (Fig. 1D). Radula uniserial, very small compared to pharynx internal volume (Fig. 1E, indicated by an arrow). Radular formula 13 × 0.1.0. Central tooth narrowly triangular, with sharp to pitted top (Fig. 1F, G, H) and up to 23 denticles that are small and irregular in size, without distinct dorsal denticle furrows, only sometimes with fine rib-like structures (Fig. 1H).

Reproductive system diaulic (Fig. 4A). Ampulla moderate in size (Fig. 4A, am). Vas deferens short, without distinct prostatic portion (Fig. 4A, vd), penial sheath widened (Fig. 4A, psh), penis unarmed, with elevations (Fig. 4A, p). Single proximal receptaculum seminis very large, oval (Fig. 4A, rsp).

##### Biology.

Subtidal, highly cryptic on *Pennariadisticha* hydroids in soft sediment habitats beyond the coral reef or on the same hydroids at the reef edge (Fig. 1A, B). Egg mass is a long narrow ribbon, white, laid directly onto host hydroids (Fig. 1B).

##### Distribution.

Presently found only at Koh Samaesan, Thailand, but expected to be found in neighbouring regions of the Indo-West Pacific.

##### Remarks.

Thai specimens show closeness to the type species of the genus *Myjalongicornis* from Ambon (Indonesia) in such features as the apically widened cerata, only a single pair of anterior cerata, acleioproctic anus, winged jaws, and small uniserial radula. Therefore, studying these specimens allows us to reveal the phylogenetic and taxonomic position of the genus *Myja* via both morphological and molecular means. However, while M.cf.longicornis from Thailand is similar to the type species of the genus *Myja*, *M.longicornis* from the type locality in Ambon as described in the original description by Bergh (1896), there are differences in several external and internal characters which do not allow us to identify the Thai material as *M.longicornis* and Bergh’s figures are reproduced here (Fig. 1I, H). We therefore record here the specimens from Thailand as M.cf.longicornis. The distinguishing features of M.cf.longicornis are predominantly greyish without the green digestive gland branches both in the body and in the cerata, as was clearly indicated for *M.longicornis* in the original description (Bergh, 1896: 389, 390). It has a reddish and not brown-chocolate basal spot at anterior pair of cerata, and similar reddish (and not brown) pigment at ceratal apices. Furthermore, the radula of *M.longicornis* as depicted in Bergh (1896; reproduced here Fig. 1J) has more distinct lateral denticles, which are lower in number (10), compared to M.cf.longicornis (at least 23) (see Fig. 1H). We suspect that there is hidden species diversity in the genus *Myja* of the Indo-West Pacific. Specimens collected in 2016 reveal the presence of at least two more species of the genus *Myja*, which differ from M.cf.longicornis based on morphological and molecular data and from *M.longicornis* according to the morphological data, are described as new to science, *Myjakarin* sp. n. (see Fig. 2) and *Myjahyotan* sp. n. (see Fig. 3). Minimum uncorrected p-distances of the COI marker which separate M.cf.longicornis from *M.karin* sp. n. are 11.9%. Minimum uncorrected p-distances of the 16S marker which separate M.cf.longicornis from *M.karin* sp. n. are 3.71% and from *M.hyotan* sp. n. are 2.55%. Minimum uncorrected p-distances of the H3 marker which separate M.cf.longicornis from *M.karin* sp. n. are 4.28% and from *M.hyotan* sp. n. are 3.36%, whereas p-distances between the two specimens of M.cf.longicornis for COI, 16S, and H3 markers are 0.2%, 0%, and 0% respectively.

#### Myja karin sp. n.

Taxon classificationAnimaliaNudibranchiaTergipedidae

http://zoobank.org/789A7CE3-31D2-457A-9DE0-9D1C4878C9F4

Figs 2 , 4B , 5

##### Type material.

Holotype, ZMMU Op-610, ca. 12 mm long (alive), Japan, Osezaki, 10 Sept 2016, depth 7–15 m, stones, rocks, hydroids, collector Tatiana Korshunova, Alexander Martynov. Paratype, ZMMU Op-611, Japan, Uchiura, 09 Sept 2016 depth 20 m, collector Hiroshi Takashige.

##### Type locality.

Japan.

##### Etymology.

In honour of Karin Fletcher (Port Orchard, Washington), who has made considerable recent efforts in uncovering hidden diversity and understanding of the nudibranch fauna of the NE Pacific.

##### Diagnosis.

Up to ten ceratal rows, ground colour translucent greyish, ceratal cores light to dark greyish, ceratal tops dull reddish, apices with white spot, anterior cerata with brownish basal spot, no sparse white spots in the first half of the dorsal part, white gonad spherules moderately dense, cerata moderately widened at top without smaller separate cupola-shaped tip, central tooth narrowly triangular with very sharp non-pitted top and numerous lateral denticles, up to 20–30 small irregular in size denticles, very distinct ridges and furrows on the teeth surface, no accessory penial gland, penis unarmed.

##### Description.

Body very elongate, holotype ca. 12 mm alive (Fig. 2 A–D). Rhinophores ca. 1.5 times longer than oral tentacles, smooth. Dorsal papillae cylindrical to spindle-shaped, forming nine or ten ceratal rows along dorsal edges. Apices of papillae form moderate oval swellings, without cupola-shaped appendage (Fig. 2E). Notal edge absent. Anal opening acleioproctic on right side before first posterior ceratal rows. Reproductive openings lateral, below first anterior and second posterior rows of cerata. Ground colour translucent greyish. Oral tentacles and rhinophores with scattered opaque white dots. On head after oral tentacles shines a small pinkish area, lateral sides of head with thin streaks of brown-orange pigment. Opaque white spots in anterior part of the body behind rhinophores absent. Between rhinophores shines a large brownish area. Digestive gland in the cerata (ceratal cores) whitish to light creamy and light greyish (basal parts can be very pale greenish), digestive gland in upper part of cerata with dull pinkish-brownish internal spot, apices mostly translucent with small white band at very tip. Anterior cerata with prominent brownish basal spot. A spot similar in colour, but duller brownish and smaller in size, may occur at basal part of other cerata. Central branches of digestive gland shine through dorsal part of body and are brownish with few greyish parts. Numerous, moderately dense, small, white gonads appeared as white spherules that shine through dorsal surface. Jaws broadly triangular with prominent anterior wings, masticatory borders smooth (Fig. 2I, J). Radula uniserial, very small compared to the pharynx internal volume (Fig. 2K). Radular formula 17 × 0.1.0. Central tooth narrowly triangular with very sharp top and up to ca. 20–30 (and probably more) small denticles, irregular in size (Fig. 2L–N), often hard to delineate with very distinct dorsal denticle furrows and fine rib-like structures (Fig. 2M, N).

Reproductive system diaulic (Fig. 4B). Ampulla moderate in size, slightly widened in the middle (Fig. 4B, am). Vas deferens short, without distinct prostatic portion (Fig. 4B, vd), penial sheath widened (Fig. 4B, psh), penis unarmed, with at least two unequal elevations (Fig. 4B, p). Single proximal receptaculum seminis very large, elongated (Fig. 4B, rsp).

##### Biology.

Subtidal, on stony and rocky area with the hydroids *Pennaria* sp. (Fig. 2F). Egg mass is a long, convoluted ribbon (Fig. 2D). Veligers are planktonic, with turbospiral shell (Fig. 2G, H).

##### Distribution.

Central parts of the Pacific coast of the main Japanese island of Honshu; potentially can occur at least at the southern parts of Honshu and Kyushu.

##### Remarks.

The type species of the genus *Myja*, *M.longicornis*, is similar externally to *Myjakarin* sp. n. by presence of brown anterior basal ceratal spots, bur readily distinguished by predominantly brownish-pinkish, and not green, main branches of digestive gland, and also by white to greyish rather than green ceratal cores (Fig. 2). Bergh (1896; see Fig. 1) also reported seven pairs of cerata for three large specimens (up to 15 mm alive, 9.5–10 mm fixed), whereas *M.karin* sp. n. of ca. 12 mm length alive has up to ten cerata (Fig. 2A–C). Furthermore the radula of *M.longicornis* as depicted in Bergh (1896) has a sharp apical part (Fig. 1J), somewhat like in *M.karin* sp. n., but there are considerably fewer lateral denticles [6–7 on the figure in Bergh (1896), up to ten in the description in Bergh (1896)], compared to *M.karin* sp. n. with up to 20–30 lateral denticles at least (Fig. 2M, N). Myjacf.longicornis from Thailand differs from *Myjakarin* sp. n. by its reddish and not brownish basal anterior ceratal spots and very considerably by the morphology of its radula (compare Fig. 1F–H with Fig. 2L–N). One more new species of the genus *Myja*, *Myjahyotan* sp. n. described below from Japanese waters, differs from *Myjakarin* sp. n. by details of body colour, radular characteristics (see detailed remarks below and Table 2 for details), and according to molecular phylogenetic data (Fig. 5). Minimum uncorrected p-distances of the COI marker which separate *M.karin* sp. n. from M.cf.longicornis are 11.9%. Minimum uncorrected p-distances of the 16S marker which separate the *M.karin* sp. n. from M.cf.longicornis are 3.71% and from *M.hyotan* sp. n. are 4.41%. Minimum uncorrected p-distances of the H3 marker which separate *M.karin* sp. n. from M.cf.longicornis is 4.28% and from *M.hyotan* sp. n. is 3.98%. P-distances between the two specimens of *M.karin* sp. n. for the COI, 16S, and H3 markers are 0.5%, 0.7%, and 0% respectively.

**Table 2.** Morphological comparison of the species belonging to the genus *Myja*.

	Maximum length alive	Colour of central branches of digestive gland	Colour of digestive branches in cerata	Colour of internal spot of digestive gland in upper part of cerata	Colour of large spot of digestive glad at the base of right anterior cerata	Radula teeth
M. cf. longicornis	10 mm	Greyish	Greyish	Reddish	Reddish	With sharp to pitted central cusp and numerous indistinct, irregularly placed lateral denticles (up to at least 23), no furrows and ribs
*M.karin* sp. n.	12 mm	Brownish, few pieces greyish	Greyish to whitish	Pinkish-brownish	Brownish	With very sharp central cusp and up to ca. 20–30 of small irregular in size denticles, often hard to delineate, with deep furrows and fine rib-like structures
*M.hyotan* sp. n.	20 mm	Dark brownish	Whitish to light cream	Dark brownish	Dark brownish	With sharp, largely non-pitted cusp and up to ca. 10 (often no more than 5 denticles) relatively distinct small denticles in anterior part of radula to completely smooth or with very indistinct denticles in posterior part of radula
*M.longicornis* Bergh, 1896	15 mm	Green	Green	Brown	“Brown-chocolate”	With sharp pointed central cusp and 6–10 distinct regularly placed lateral denticles

#### Myja hyotan sp. n.

Taxon classificationAnimaliaNudibranchiaTergipedidae

http://zoobank.org/995BFF5F-198C-4C1E-97CB-A018B51B8876

Figs 3 , 4C , 5

Eubranchus sp. 7 Nakano, 2004: 244.

##### Type material.

Holotype, ZMMU Op-612, ca. 20 mm long alive, Japan, Osezaki, 10 Sept 2016, depth 7–15 m, stones, rocks, hydroids, collector Tatiana Korshunova, Alexander Martynov.

##### Type locality.

Japan, Osezaki.

##### Etymology.

After the Japanese name hyōtan (瓢箪, ヒョウタン) for the calabash *Lagenariasiceraria*, the fruits of which are very similar in shape to the peculiar cupola-shaped tip of cerata of this new *Myja* species.

##### Diagnosis.

Up to eight ceratal rows, ground colour translucent greyish, ceratal cores white to dark greyish, ceratal tops dull reddish, no apical white spot, anterior cerata with prominent dark brownish basal spot, sparse white spots in the first half of the dorsal part, white gonad spherules very dense, cerata considerably widened at top with smaller separate cupola-shaped tip, central tooth narrowly triangular with largely non-pitted top and only few denticles, up to ten small denticles, irregular in size; no accessory penial gland, penis unarmed.

##### Description.

Body very elongate, holotype ca. 20 mm (alive, Fig. 3A–C). Rhinophores up to ca. two times longer than oral tentacles, smooth. Dorsal papillae cylindrical and then rapidly widened at the top, forming up to eight ceratal rows along dorsal edges. Apices of papillae considerably widened with smaller separate cupola-shaped tip appendage (Fig. 3D). Notal edge absent. Anal opening acleioproctic on right side before first posterior ceratal rows. Reproductive openings lateral, below first anterior and second posterior rows of cerata. Ground colour translucent greyish, but because of presence of numerous, very densely placed gonad spherules, appears as rather opaque white. Oral tentacles and rhinophores with few scattered opaque white dots. On head after oral tentacles to in between of rhinophores shines a pinkish area, lateral sides of head without thin streaks of brown-orange pigment. Opaque white in anterior part of the body after rhinophores. Between rhinophores shines a large brownish area. Digestive gland in the cerata (ceratal cores) whitish to light creamy, digestive gland in upper part of cerata with dark brownish internal spot, apices mostly translucent, without small white band at very tip. Anterior cerata with prominent dark brownish basal spot. A spot similar in colour, but duller brown and smaller in size, may occur at basal part of other cerata. Central branches of digestive gland shining through dorsal part of body are dark brownish. Numerous, very dense small white gonads appear as white spherules that shine through dorsal surface and create a rather opaque white dorsal appearance. Jaws broadly triangular with prominent anterior wings, masticatory borders smooth (Fig. 3F, G). Radula uniserial, very small compared to internal volume of the pharynx (Fig. 3H). Radular formula 15 × 0.1.0. Central tooth narrowly triangular with sharp or rarely pitted top and up to ca. ten (often no more than five) relatively distinct small denticles in anterior part of radula (Fig. 3I–K) to completely smooth or with very indistinct denticles in posterior part of radula (Fig. 3K). Few teeth in posterior part of radula may have pitted top (Fig. 3K)

Reproductive system diaulic (Fig. 4C). Ampulla moderate in size, slightly widened in the middle (Fig. 4C, am). Vas deferens short, without distinct prostatic portion (Fig. 4C, vd), penial sheath widened (Fig. 4C, psh), penis unarmed, with elevations (Fig. 4C, p). Single proximal receptaculum seminis very large, pyriform (Fig. 4C, rsp).

##### Biology.

Subtidal, on stony and rocky area with hydroids *Pennaria* sp. (Fig. 3E). No data on egg mass so far.

##### Distribution.

Central parts of the Pacific coast of main Japanese island Honshu; potentially can occur at the southern parts of Honshu and Kyushu.

##### Remarks.

The type species of the genus *Myja*, *M.longicornis* is somewhat similar externally to *Myjahyotan* sp. n. by presence of brown anterior basal ceratal spot, but it is readily distinguished by dark brown and not green main branches of digestive gland, and also by the white rather than green ceratal cores. Another notable difference between the type species and all other species described here from *M.hyotan* sp. n. is the very densely placed white spherules of the gonad that shine through the dorsal body and appear as opaque white in *M.hyotan*. The shape of the cerata in *M.hyotan* sp. n. also readily differentiates it from *M.longicornis*, M.cf.longicornis, and *M.karin* sp. n. with the presence of an additional, separate, cupola-shaped top chamber in the ceratal apices (Fig. 3D). Furthermore, the radula of *M.longicornis* as depicted in Bergh (1896; reproduced here Fig. 1J) has a sharp apical part, somewhat similar to that of *M.hyotan* sp. n., but the denticles in *M.longicornis* are much more distinct, compared to *M.hyotan* sp. n., in which in most of the radula (except few anterior most teeth) has lateral denticles either absent or very indistinct (Fig. 3I, K). Myjacf.longicornis differs from *M.hyotan* sp. n. by the reddish and not brownish basal anterior ceratal spot and very considerably by the morphology of radula (compare Fig. 1F–H and Fig. 3I–K). *Myjahyotan* sp. n. differs from the other new species of the genus *Myja* from Japan, *M.karin* sp. n., by the shape of the cerata (including cupola-shaped separate tip), very dense white spherules of gonads, presence of white spots on the dorsal part behind the rhinophores, by radular characteristics (*M.hyotan* sp. n. fully devoid of peculiar furrows and ridges on the teeth as present in *M.karin* sp. n., and many teeth of *M.hyotan* sp. n. almost smooth, without denticles) (see Table 2 for details) and according to the molecular phylogenetic data (Fig. 5). Minimum uncorrected p-distances of the 16S marker which separate the *M.hyotan* sp. n. from M.cf.longicornis is 2.55% and from *M.karin* sp. n. is 4.41%. Minimum uncorrected p-distances of the H3 marker which separate *M.hyotan* sp. n. from M.cf.longicornis is 3.36% and from *M.karin* sp. n. is 3.98%.

## Discussion

The phylogeny and taxonomy of the Aeolidacea have been the subject of numerous recent studies (e.g., Millen and Hermosillo 2012; Carmona et al. 2013; Padula et al. 2014; Kienberger et al. 2016; Korshunova et al. 2017a; Goodheart et al. 2018). The genus *Myja* is unique among both traditional Facelinidae and all known Aeolidacea families by having a combination of tergipedid- or eubranchid-like external appearance with just a single row per side of anterior cerata (with functional cnidosacs) and an acleioproctic anus, facelinid-like winged jaws, the absence of a supplementary gland in the reproductive system, and a unique very small radula. Initially, Bergh (1896) placed the genus *Myja* in the family Tergipedidae probably because of the presence of an acleioproctic anus in combination with few cerata per row and the uniserial radula, despite the absence of the supplementary gland in the reproductive system and shape of the jaws considerably differing from tergipedids and indicating placement within the Facelinidae. Furthermore, together with the first description of the genus *Myja*, Bergh (1896) described a new genus and species *Ennoiabriareus* Bergh, 1896 (also within the family Tergipedidae) which was later transferred to the traditional facelinid genus *Phyllodesmium* using only morphological data (Rudman 1991). Thus, in 1896, it was potentially possible to suggest facelinid affinity of the genus *Myja* using available morphological characters. Despite this, during the past century the genus *Myja* and the sole species *M.longicornis* has been included into a few classification reviews (e.g., Thiele 1931; Parker 1982; Vaught 1989) and colour guides and other publications (e.g., Marcus 1965; Cobb and Willan 2006; Coleman 2008) within the family Tergipedidae only. Recently Gosliner et al. (2015: 336) placed *Myja* as an “undetermined family”, but no trees or molecular analyses have been presented since that publication. In the recent edition of the colour guide on the Japanese sea slugs *Myja* was also placed in an undetermined family (Nakano 2018). We have conducted this study since 2016 (TK and AM collected *Myja* specimens during research trip to Osezaki, Japan) and while our study was at a final stage, an abstract of a conference mentioning the genus *Myja* has appeared (Ekimova et. al. 2018). Thus, the taxonomic position of the genus *Myja* until recently was not evaluated or challenged in a journal or book publication since Bergh's (1896) first description.

Our present molecular data and morphological analysis of the genus *Myja* clearly shows that previous morphological assessment was incorrect. Our new data places the genus *Myja* as phylogenetically related not just to the Facelinidae s. l., but to the group of Facelinidae s. str. close to the type species of the genus *Facelina* (see below for details). However, in strong contrast to molecular data, the external morphological characters of the species of the genus *Myja* are highly unusual and resemble those of members of the family Eubranchidae, and those of the Tergipedidae (genus *Tergipes*), but are drastically different from any described genera of the family Facelinidae. For example, the external similarity the species described here *Myjahyotan* sp. n. to some members of the family Eubranchidae is so striking that it was previously identified as *Eubranchus* sp. 7 (see Nakano 2004: 244). Furthermore, Bergh (1896) has compared the radula of the genus *Myja* with that of the genus *Calma* (known at that time under the name *Forestia* Trinchese, 1881). While particular radular teeth of the highly unusual partially fused radula of the genus *Calma* have showed some superficial similarities (Fig. 1P) to some of the species of the genus *Myja*, e.g., to the newly described *M.hyotan* (see Fig. 3K), it is not similar either to the type species of *Myja* as described in Bergh (1896) (see Fig. 1J) or to *M.karin* sp. n., described above (Fig. 2M, N). According to the recent molecular phylogenetic data (Korshunova et al. 2018a), the genus *Calma* and family Calmidae are not related to *Myja*.

The long taxonomic problem of the classification of the aeolidacean nudibranch family Facelinidae (e.g., Risso-Dominguez 1962, 1964; Schmekel 1966, 1967; Edmunds 1970; Miller 1974; Picton 1979; Rudman 1980, 1991; Gosliner and Behrens 1986; Hirano 1999; Millen and Hermosillo 2012; Churchill et al. 2014; Goodheart et al. 2017; and others) is one of the best cases to demonstrate the failure of a purely molecular phylogenetic approach (e.g., Carmona et al. 2013) to build a classification. The oldest name for the assemblage of facelinid families is Glaucidae Gray, 1827 and Facelinidae itself was proposed by Bergh much later in 1889 (MolluscaBase 2018a, b), but all facelinid diversity had been suggested to be merged under the name Glaucidae (Miller, 1974). Recently Goodheart et al. (2017: 10) indicated that because of paraphyly of traditional Facelinidae “…until a member of the genus *Facelina* (the type genus for this family) is included in the analyses (ideally the type taxon *Facelinaauriculata*), it is impossible to say which clade should receive the Facelinidae designation.” In the present study we fully meet these requirements. Obtained here for the first time is molecular data for *Facelinaauriculata* (Müller, 1776) (= *Facelinacoronata* (Forbes & Goodsir, 1839)) and this is included with data from other *Facelina* species in the molecular phylogenetic analysis (Fig. 5). The analysis has placed at least four species of the genus *Facelina* into a well-supported clade together with the type species *F.auriculata* (Fig. 5).

Originally, the type species of the genus *Facelina* is *F.coronata* (see Alder and Hancock 1845–1855: xxii). The older name *F.auriculata* was restored for this species by Odhner (1939), though he mistakenly synonymised *Eolisdrummondi* Thompson, 1844, and hence *Eoliscurta* Alder & Hancock, 1843 (currently both are junior synonyms of *F.bostoniensis* (Couthouy, 1838), see Thompson and Brown 1984)) with *F.auriculata*. Thompson (1976) used the name *F.auriculata* in the subspecies combination *F.auriculatacoronata*, but later he declined to apply the name *F.auriculata* as senior synonym of *F.coronata* (Thompson & Brown, 1984) because of putatively uncertain separation from *F.bostoniensis*. However, the figure of “*Doris*” *auriculata* as depicted in Müller et al. (1806) clearly shows separated clusters of short cerata and thus cannot be referred to *F.bostoniensis* (including *F.curta*) with overlapping rows of long cerata. The work of Müller et al. (1806) is an integral part of the original “Zoologiae Danicae…” (Müller 1776) and has continuing volume numeration with the latter. Therefore, figure 1 on the plate CXXXVIII of “*Doris*” *auriculata* in Müller et al. (1806) belongs to the original description of *F.auriculata*. Odhner (1939) also mentioned the similarity of Müller’s figure of “*Doris*” *auriculata* to *F.coronata*. Importantly, both Müller (1776: 229) and Müller et al. (1806: 21) gave reference to an older work by Hans Ström as “A. Havn., 10. p.16. t. 5. fig. 6” as a basis for their descriptions while describing “*Doris*” *auriculata*. According to Müller (1776: X) “A. Havn.” is an acronym for the journal “Det Kiobenhavnske Selskabs Skrifter” (= Skrifter som udi det Kiøbenhavnske Selskab) which in Latin is “Actis Societatis Historiae Naturalis Havniensis”. We have thus explored the work by Ström (1765–1769: 16) and found a fairly detailed description (including a figure) under the non-binomial name “Thetys auriculis duabus, pilis dorsi mollibus, fasciculatis, erectis” in Latin. Among other characters Ström mentioned “…the whole body colour is white and glossy (blank), the tassel-shaped lungs [= cerata] purple-red with white tips....” (“at hele Kroppens Farve er hvid og blank, men de Qvast- [= modern Danish ”kvast”] dannede Lunger Purpur-røde med hvide Spidser...)” (Ström 1765–1769: 16). This colour description almost perfectly fits the colour pattern of the species that we currently accept under the name *F.auriculata*. Furthermore, in figure 6 in Ström (1765–1769: tab. V) there are clear ceratal clusters, oral tentacles longer than the rhinophores (which are likely perfoliated), and anterior foot corners. Thus, both colour and external characters of Ström’s description of “Thetys auriculis duabus…”, that becomes the basis for Müller’s (1776: 229) description of “*D.*” *auriculata*, agree very well with the characters of the currently recognized *Facelinaauriculata*. Apparently Thompson did not check the original description of Hans Ström, because the doubts about synonymy of *F.coronata* with *F.auriculata* as expressed in the work of Thompson and Brown (1984: 150-1) would have been unnecessary. According to ICZN (1999) articles 11.4 and 11.5 Müller (1776: 229) thus made the non-binomial name of Ström the fully valid and available binomial name “*D.*” *auriculata* and provided the bibliographic reference to Ström's (1765–1769) work. Picton and Morrow (1994) started the current usage of the name *F.auriculata* and Picton (2001) published the original figure of “*Doris*” *auriculata* from Müller et al. (1806) and further provided arguments for the validity of *F.auriculata*. Here we present for the first time the pre-binomial history of that species and confirm that Ström’s and Müller’s descriptions of “*D.*” *auriculata* are fully concordant with the current understanding of *F.auriculata*. However, to avoid potential taxonomic problems caused by hitherto unrecognized hidden diversity within *Facelina* s. str. and taking into consideration the complex taxonomic history of the species *F.auriculata* (e.g., Odhner 1939; Lemche 1964; Thompson, 1976; Thompson and Brown 1984; Picton and Morrow 1994; present study) we designate here a neotype for *F.auriculata* (ZMMU Op-669), for which molecular data have been obtained for the first time.

The photograph of *Facelinaauriculata* on the tree (Fig. 5) is precisely the neotype designated here. The SEM of jaws and radula for *F.auriculata* are presented in this study (Fig. 1K, M–O) from another specimen of *F.auriculata* which is externally very similar to the neotype and was collected together with the neotype at the same locality and date. In another recently published paper in which the COI, 16S, and 18S genes were applied, the paraphyly of traditional Facelinidae was again shown (Goodheart et al. 2018). The paraphyletic Facelinidae clades were designated as “Facelinidae 1” and “Facelinidae 2” respectively (Goodheart et al. 2018: 12). Because in the present study we demonstrated that the type species of the genus *Facelina* is nested precisely within “Facelinidae 1” we can confidently confirm here that this group is the true Facelinidae s. str., whereas for the “Facelinidae 2”, a separate family name is necessary. The clade which contains the true *Facelina* s. str. is related to the families Favorinidae, Glaucidae s. str., and the genus *Myja*, but not to the clade of paraphyletic Facelinidae which is related to the families Aeolidiidae and Babakinidae (Fig. 5). By this, it is possible to confirm the phylogenetic placement of Facelinidae s. str. (including the type species *F.auriculata*), and state that the genus *Myja* is not related to a clade which contains genera *Dondice*, *Godiva*, *Hermissenda*, *Phyllodesmium*, and others (see Fig. 5).

While Glaucidae is phylogenetically (Fig. 5) related to the core group of Facelinidae s. str., it has a profoundly modified aberrant external morphology that has adapted it to an exclusively pelagic lifestyle compared to the exclusively benthic facelinid family group. Internally however, the Glaucidae appear to conform to the traditional Facelinidae (Miller 1974). According to the molecular data, the genus *Myja* is closest to the Facelinidae s. str., and particularly to the putatively paraphyletic genus *Cratena* (Fig. 5). However, morphologically (and hence, ontogenetically and epigenetically, see Korshunova et al. 2017c) the genus *Myja* differs from the Facelinidae s. str., thus suggesting potential separation of the genus *Myja* into a new family. Despite the proposal to merge the morphologically modified Glaucidae with the phylogenetically related facelinids (Miller 1974; Rudman 1980), this was not applied consistently (e.g., Gosliner et al. 2015). This is against the priority principle as described by the ICZN (1999, article 23.1) because Facelinidae Bergh, 1889 s. str. should be considered a junior synonym of Glaucidae Gray, 1827. This fact is of crucial importance, since many researchers previously were able to recognize a small, morphologically and molecularly distinct taxonomic unit comprising the family Glaucidae, having unique morphological features despite its close relatedness to the facelinids. This challenges the still dominant perception that molecularly related but morphologically different taxa should be merged under the same taxon. Most recently, the family Favorinidae has been suggested to be restored (Goodheart et al. 2017, 2018), despite previously being almost universally considered as a synonym of the Facelinidae and that the Favorinidae is much more complicated to delineate morphologically from Facelinidae s. str. than the Glaucidae.

The family Glaucidae was not included in the analysis in Goodheart et al. (2017: 10), but the same name “Facelinidae” was instead applied for several clades, including those strongly paraphyletic ones. Recently the genus *Glaucus* was included in an analysis by Goodheart et al. (2018) and was shown as closely related to the Facelinidae s. str., thus fully corroborating our results (Fig. 5). Therefore, should these families be explicitly synonymised, as for example was done by Miller (1974), the oldest name Glaucidae (with Facelinidae s. str. at least as their junior synonym) should be utilised. That the inconsistent usage of the family name Glaucidae has also continued in recent papers, for example in Churchill et al. (2013: 2), the subfamily Glaucinae in a very narrow sense was discussed as “Glaucinae contains a single genus, *Glaucus*…” and thus the facelinid problem was not discussed, despite the mention that *Glaucus* is placed in the clade with such traditionally facelinid genera as *Favorinus* Gray, 1850, *Learchis* Bergh, 1896 and *Hermosita* Gosliner & Behrens, 1986 (Churchill et al. 2013: 4). Churchill et al. (2014: 175) later stated of the family Glaucidae “*Glaucus* is the type genus (and *G.atlanticus* the type species) of the large family Glaucidae Gray, 1827” implying that Facelinidae is included in Glaucidae as a synonym, but this was not discussed. This results in a contrast with previous morphological conclusions that Glaucidae “could be closely related to *Cuthona* (Family Tergipedidae) rather than to *Facelina* and other related groups” (Valdés and Campillo 2004: 381) but in agreement with morphological conclusions of other authors, that Glaucidae is in the same group as Facelinidae (e.g., Miller 1974; Rudman 1980). Valdés and Campillo (2004: 382) further argued that “unless the Glaucinae is, in the future, found to be much more diverse than is currently recognized, the maintenance of a single genus is sufficient to express the diversification that has taken place in this group.” The implication is that if more hidden diversity would be discovered, then the generic classification of Glaucidae should be reconsidered. Ten years later it was revealed that hidden diversity within the “*Glaucusmarginatus* group” does exist (Churchill et al. 2014). Due to the high concordance of the distinct molecular clades and morphological data, we here restore within glaucids the genus *Glaucilla* Bergh, 1861, stat. n. which clearly differs from the genus *Glaucus* by the different arrangement of the cerata in multiseriate groups, the short posterior end of the body, the different position of the nephroproct, and by the unarmed penis (Bergh 1861; Miller 1974; Valdés and Campillo 2004). Three further described species within the genus *Glaucus* s. l. (Churchill et al. 2014) are fully consistent with these differences and therefore are transferred here to the genus *Glaucilla* as follows: *Glaucillabennettae* (Churchill, Valdés, Foighil, 2014), comb. n., *Glaucillamcfarlanei* (Churchill, Valdés, Foighil, 2014), comb. n., and *Glaucillathompsoni* (Churchill, Valdés, Foighil, 2014), comb. n. The type species of genus *Glaucilla*, *Glaucillamarginata* Reinhardt in Bergh, 1864, stat. n. is therefore returned to its original combination in this work.

The present study confirms that Glaucidae and Facelinidae s. str. are closely related according to the molecular data (Fig. 5). This implies that it is understood that the current classification poorly integrates morphological and molecular information but because of the dominant taxonomic framework, a major reassessment has still not been performed. Under a lumping approach, the genus *Myja* can be included within the family Facelinidae s. str., despite considerable morphological disparity, but then it can be proposed that the family Facelinidae Bergh, 1889 should be synonymised with the family Glaucidae Gray, 1827 as the latter is phylogenetically closely related to Facelinidae and glaucids do not differ fundamentally (morphologically) from facelinids. This approach then would also make the recently restored Favorinidae (Goodheart et al. 2017) redundant. However, as has already been shown (Korshunova et al. 2017a), such a broad approach as the synonymy of Glaucidae with Facelinidae would only be the beginning of an avalanche-like potential synonymisation process of the families within the suborder Aeolidacea. For example, Babakinidae is phylogenetically related to both Aeolidiidae and Facelinidae but has a radula that is similar to Facelinidae but not to Aeolidiidae (Roller 1972, 1973; Carmona et al. 2011; Korshunova et al 2017a). The family Apataidae in turn is not related closely to the Flabellinidae, but to the superfamily Fionoidea; however, it has a triserial radula and a reproductive system that does not differ fundamentally from the family Flabellinidae. Furthermore, the family Eubranchidae has a triserial radula and a trinchesiid-like reproductive system and is phylogenetically related to both the family Apataidae and the superfamily Fionoidea. Finally, the genus *Fiona* is a complete analogue of Glaucidae as the latter has peculiar morphological adaptations to the neustonic environment and is morphologically very different from the majority of the Fionoidea by the presence of a distinct notal edge and the absence of the supplementary penial gland. It is also, however, phylogenetically closely related to the morphologically disparate Tergipedidae and Trinchesiidae (Korshunova et al. 2017b, 2018a, b). Thus, the internal groups within the suborder Aeolidacea form a very complicated morphological and molecular mosaic and under a super-lumping approach it would be unavoidable to unite all aeolidacean families into a single one. Such a decision would further raise the question of the delineation of the suborder Aeolidacea from other major nudibranch subgroups. Although the Antarctic family Notaeolidiidae have single cnidosacs in their cerata and phylogenetically appear as a basal group within Aeolidacea (Korshunova et al. 2017a; Goodheart et al. 2018), they also possess a multiserial radula similar to the dendronotacean and arminacean nudibranchs. Such a super-lumping approach thus would immediately ruin any possibility to make an integrative molecular and morphological taxonomy, because under the same family “roof” such morphologically drastically different groups as Aeolidiidae, Paracoryphellidae, or Pseudovermidae would have to be united.

For the taxonomy of the traditional family Facelinidae this means that it can be further divided into several more narrowly defined families that will integrate both morphology and molecular data instead of disintegrating it. The genus *Myja* possesses a unique combination of external and internal characters that distinguish it from any other families of the Aeolidacea (see also remarks above). Particularly, the presence of a permanent acleioproctic anus (a common feature in such families as Tergipedidae and Trinchesiidae) in combination with a small reduced radula readily differentiate the genus *Myja* from all the numerous facelinid taxa so far described. The presence of a narrow foot with a rounded anterior edge and the smooth masticatory edges of jaws in the genus *Myja* also rarely occur among facelinids. It is therefore possible that this genus should be separated into a new family to accommodate both morphological and molecular phylogenetic data in an integrative way; however, this is being left for a further study when more data on other traditional facelinids can be included. The paraphyly of the traditional facelinids indeed should be also addressed. There are two family names available for the ex-facelinid paraphyletic clade (Fig. 5) that contains several facelinid genera. One is Myrrhinidae Bergh, 1905 (Bergh 1905) and the other is Phyllodesmiidae Thiele, 1931 (originally suggested as a subfamily, Thiele 1931: 749). According to Rudman (1981) the genus *Myrrhine* Bergh, 1905 is a synonym of the genus *Phyllodesmium* and these two family names are thus referred to the same taxonomic group, but Myrrhinidae Bergh, 1905 has precedence over Phyllodesmiidae Thiele, 1931. According to the ICZN (1999) article 40.1, synonymy of the type genus in the family group does not affect validity of family-group name (if the junior family name is not in prevailing usage and the senior name was not substituted before 1961). Neither Myrrhinidae Bergh, 1905 (Bergh 1905) nor Phyllodesmiidae Thiele, 1931 were ever in prevailing usage. Both these family names were rarely used (e.g., Risso-Dominguez 1964), never synonymised with each other when listed in reviews (e.g., Thiele 1931; Parker 1982; Vaught 1989), but only with Facelinidae s. l. or Glaucidae s. l. (e.g., Rudman 1981). Therefore, we apply provision of the ICZN article 40.1 and hereby restore Myrrhinidae Bergh, 1905 (= Phyllodesmiidae Thiele, 1931) for the ex-facelinid paraphyletic group of genera including *Phyllodesmium* (= *Myrrhine*), *Hermissenda*, *Dondice*, and *Godiva* according to the priority principle. Usage of the resurrected family name Myrrhinidae Bergh, 1905 can preliminarily solve the problem of paraphyly of the traditional Facelinidae. However, the genus *Phyllodesmium* is very different indeed from the other members of this clade (e.g., absence of cnidosacs, modified cerata) such as *Hermissenda*, *Dondice*, and *Godiva* and thus does not fulfil the criteria for morphological and molecular consistency. The taxon sampling in the present study is not targeted to be exhaustive, and there are some more potential paraphyletic events also within the superclade of “Facelinidae” s. str. (see Fig. 5). Therefore, we leave further narrow-taxon based delimitation of these paraphyletic facelinid groups to a later study.

## Supplementary Material

XML Treatment for Myja

XML Treatment for Myja cf. longicornis

XML Treatment for Myja karin

XML Treatment for Myja hyotan
